# Supplementary material for: Treatment outcome of patients with prosthetic stuck valves at the Cardiac Center of Ethiopia
Source: PLoS One. 2023 Apr 20;18(4):e0284652. doi: 10.1371/journal.pone.0284652 (PMC10118175; doi:10.1371/journal.pone.0284652)
Supplement: Attachment — Submitted filename: Response to Reviewers.docx [file pone.0284652.s002.docx]

**Point_by_point Response**

Dear Editor and reviewers,

Thank you so much for reviewing our manuscript. Below is the point-by-point response that we provided to your queries/comments.

**Editor’s comments**

Regarding Journal Requirements:

1. Make sure journal requirements are addressed:

***Response:*** *Thank you. We have ensured that the manuscript meets PLOS ONE’S requirement with regard to template style and file naming.*

2. Revise Competing Interests section:

***Response:*** *As per your request, we have revised the competing interest statement as follows and the same statement is also mentioned in the revised* ***“cover letter”****. “*I have read the journal's policy and one of the authors of this manuscript have the following competing interest: *Dr Atnafu Mekonnen Tekleab is serving as Academic Editor of PLOS ONE Journal. However, this does not alter our adherence to PLOS ONE policies on sharing data and materials.”*

3. Include Data availability statement:

**Response:** As per your request, we have uploaded the anonymized data set as “Supporting Information Files”.

**Additional Editor Comments:**

**Comment:** In addition to responding to the queries of the reviewers, the authors are requested to highlight if there is any new contribution, this study offers or if there is any specific regional perspective this study brings about.

***Response:*** *Thank you for the comment. We have mentioned the implication of our finding by highlighting the higher success rate of fibrinolysis therapy and its implication in resource limited settings such as ours where access to open-heart surgery is limited. (Page-11, lines 204-212).*

**Reviewers’ comments**

**Reviewer #1:** The sample volume is small and single Center as stated by the authors themselves.

**Comment:** The total no of patients , who had valve surgery during the study period can be provided, to calculate the incidence and prevalence of prosthetic valve thrombosis

***Response:*** *We have included the following statement in the revised manuscript: “During the same period, a total of 133 patients underwent prosthetic valve replacement surgery making the prevalence of PVT to be 8.3%.” (page-7, lines 135-136)*

**Comment:** The clinical characteristics and pattern of anticoagulation and the INR and TTR status of all the patients who had valve replacements during this period, will be of great value in statistical analysis and to identify the predictors of PVT

***Response:*** *Thank you for the comment. However, we don’t have the full data regarding INR and TTR for most of the patients who underwent mechanical valve replacement surgery during the time our study subjects developed stuck valve. This is because stuck valve is an event which happens over time and hence it is not possible to get INR value of all other patients when one patient develops stuck valve. Also, we used retrospective research design which makes it impossible to get such data sets.*

**Reviewer #2: Introduction:**

**Comment:** P3 Line 6-7: The author mentions surgical approach, fibrinolysis, and heparin treatment are considered as treatments of choice. As per guidelines, for patients with a thrombosed left-sided mechanical prosthetic heart valve who present with symptoms of valve obstruction, urgent initial treatment with either slow-infusion, low- dose fibrinolytic therapy or emergency surgery is recommended. Heparinisation is not a treatment of choice.

***Response:*** *We agree with the reviewer’s comment and such is the recommendation by the 2020 ACC/AHA guideline for “patient with thrombosed left sided mechanical valve who present with* ***symptoms of valve obstruction****”. However, heparinisation can also be used in* ***“non-obstructive left side PVT”*** *as it is mentioned in reference No.2 and that is why we mentioned so in the introduction section.*

**Methods:**

**Comment:** P3 Line 27-29: The following phrase need not be mentioned in study setting “ Following the valve replacement surgery, patients might develop stuck valve and present with an emergency condition”

***Response:*** *Thank for the comment. We accepted the comment and removed the phrase.*

**Comment:** Study design and study period:
P4 Line 2: The actual study design should be detailed.

**Response:** Comment accepted and we have included the following statement in the revised manuscript: *“A retrospective institutional based cross-sectional study design was used and patients who developed stuck valve and who were treated in the center between the periods July 2017 to March 2022 were included in the study.”* (Page-4, lines 79-82).
Data collection technique:

**Comment:** P4 Line 6: The inclusion criteria not properly delineated- whether all the consecutive patients with stuck valve during the study period were included. Any exclusion criteria to be added. “Information that could help authors identify individual patients were not included in the data collection”- requires clarity

**Response:** we have mentioned the inclusion criteria as *“All patients who were diagnosed to have prosthetic stuck valve during the mentioned period were included in the study.”* (Page 5, lines 84-85). We haven’t used the term **“Consecutive”** in order to avoid confusion since the term **“consecutive”** refers to prospective study design which is not the case in our study. Also, we have defined what “***Prosthetic valve****”* and “*prosthetic stuck valve*” mean (page-5, lines 92-98). We included all patients who fulfilled the inclusion criteria and no case was excluded as long as the inclusion criteria was met.

We included the statement *“Information that could help authors identify individual patients were not included in the data collection*” in the manuscript after we were requested to include such statement by the **journal during manuscript submission** as part of ethics statement and also to declare that we have maintained patient confidentiality.

**Comment:** P4 Line 8-12: The author mentions the data collected in the given lines.
Has the data on immediate post operative trans prosthetic valvular gradient or the gradients recorded prior to the PVT during the previous routine visits collected?
Any pre study period complications (bleeding or thrombotic) available for patients who had prosthetic valve implanted prior to the study period?

**Response:** Thank you for the comment. We haven’t collected *“the immediate post-operative trans-prosthetic gradient and the trans-prosthetic gradient during the previous routine visits”* since such data is not related to our study objective. As we have mentioned in our inclusion criteria, only those patients who were diagnosed to have stuck valve during a follow up visit were included in the study.

Also, we haven’t studied *“pre-study period complications (bleeding or thrombotic) events for patients who had prosthetic valve*” since our study objective was only “*to assess the treatment outcome of patients who had Prosthetic Valve Thrombosis*”.

**Comment:** P4 Line 10: The data on anticoagulant adherence is collected but the type of anticoagulant and the dosage, the patient is on, has not been mentioned? Were all the patient on warfarin?

***Response:*** *Yes, all the patients were taking only warfarin and we have mentioned that in the revised manuscript (page 7, line 146). We have stated that only five of them had INR value in the recommended range (page-7, lines 146-147) and we don’t think it is important to mention the dose of warfarin each patient was taking.*

**Comment:** P4 Line 11: Instead of thrombolytic treatment, interventions done can be mentioned since not all the patients were thrombolysed.

***Response:*** *Thank for the comment. We have mentioned the details of the kind of intervention that was done for each patient on page 7, lines 158-167 and also in Table 1 (Thrombolysis, surgery, heparinisation).*

**Comment:** P4 Line 15-18: Was the study entirely based on clinical and echocardiographic criteria? Was fluoroscopic criteria included in the study?

***Response:*** *As we stated on page-5, lines 92-98, the study was based on clinical and echocardiographic criteria. Additionally, echocardiographic criteria alone was used only for patients who were clinically asymptomatic but had immobile prosthetic valve disc seen on echo. However, there were only six patients who underwent fluoroscopic study in addition to the echo study and we gathered their pre-treatment fluoroscopic findings (page-7, line 153-157).*

**Comment:** What does the author mean by acute hemodynamic instability as the clinical criteria- the author also mentions “four patients, the stuck valve was detected incidentally when they came for the regular follow-up” (P6 Line 6-7). Kindly clarify the definition for stuck valve.

***Response:*** *Thank you for the comment. We have stated definition of “stuck valve” on page-5, lines 92-98. By “Acute hemodynamic instability as clinical criteria” we mean when patients had symptoms of heart failure due to the obstructive prosthetic stuck valve. Obstructive stuck valve is typically associated with overt heart failure, whereas non‐obstructive PVT is often an incidental finding. We have cited reference No. 2 which says patients can present either with hemodynamic instability or with sub-clinical condition (incidental detection) depending on the presence or absence of obstruction.*

**Comment:** The patient symptoms on the basis of NHYA is not mentioned.

**Response:** We accept the comment and we have revised the statement as follows. *“Nine patients presented with class III/IV New York Heart Association (NYHA) heart failure symptoms (median duration of symptom was three days) due to the prosthetic stuck valve and for the rest four patients, the stuck valve was detected incidentally and the patients had class I/II NYHA symptoms when they came for the regular follow-up.” (Page-7, lines 147-151).*

**Comment:** The echocardiographic criteria mentions a series of findings- where they all pre requisite for diagnosis of stuck valve in this study or a combination of the findings were alone necessary- clarification needed.

***Response:*** *Thank you for the comment*. We have stated that the presence of immobile prosthetic valve disc and elevated trans-prosthetic gradient was enough to diagnose stuck valve (page-5, lines-96-98).

**Patient management protocol for prosthetic stuck valve:**
**Comment:** P4 Line 20: The author mentions that this is the protocol for patients with obstructive thrombotic prosthetic valve. In the previous section while defining stuck prosthetic valve it is mentioned with or without obstruction(P4 Line 18). Is there different protocol for patients without obstruction? What is their symptom profile?

***Response:*** *Thank you for the comment. We have mentioned the patient management protocol for patients with stuck valve without obstruction/mild symptoms on page-6, lines 116-119.*

**Comment:** P4 Line 27: Patient will be assessed for an increased risk of bleeding- Kindly mention what criteria/ scoring was used to assess the bleeding risk.

***Response:*** Comment accepted and we have included the following statement: *…(Such as low platelet count, presence of bleeding diathesis and INR value above the target range)...* (page-5, line 106)

**Comment:** P4 Line 28-29: Mentions only the protocol for thrombolysis with streptokinase. “Ten of them received streptokinase and the remaining one patient received Tenecteplase” What was the protocol used for TNK managed patient?

**Response:** Comment accepted and we have included the following statement: *“We use Tenecteplase when patient has previous exposure to streptokinase (in the past one year) in order to avoid hypersensitivity reaction to streptokinase re-administration. Additionally, tenecteplase is more expensive in our setting and not routinely available for first line use.”* (Page-6, lines 119-122)

**Comment:** P5 Line 6: “Redo valve surgery will be offered if there is no response to the thrombolytic therapy” Is this after 72 hrs of thrombolytic therapy. Kindly clarify

**Response:** Thank you. We have included the following phrase to clarify it. “…*if no response to the thrombolytic therapy administered for 72 hrs” (page-6, line 115).*

**Comment:** P5 Line 9: “At the end, cine fluoroscopy will be done”. Is fluoroscopy done in all cases?

***Response:*** *Thank you. We have stated that Cine fluoroscopy was done only for six patients (page-8, lines 154-156) which was dictated by patients’ hemodynamic status and occasionally by the resource limitation in the center.*

**Results:**
**Comment:** P5 Line 27-28: “However, only one patient had the prosthetic stuck valve at the aortic valve position” The sample size appear to be too small to comment on this.

***Response:*** *We accept the comment and that is why we did only descriptive statistics of our study population. We haven’t done analytical statistical tests which need larger sample size to better estimate effect size.*

**Comment:** P6 Line 4-7: “Nine patients presented with symptoms of heart failure (median duration was three days) due to the prosthetic stuck valve and for the rest four patients, the stuck valve was detected incidentally when they came for the regular follow-up”
Does this mean that the incidentally diagnosed patients were also in acute hemodynamic compromise as mentioned in the definition of stuck prosthetic valve?

***Response:*** *Thank you for the comment. As we have stated on page-5, lines 96-98 (definition of stuck valve) and page-7, lines 148-150), patients who had echo features of stuck valve while they were clinically asymptomatic were the ones who were labeled as “incidentally diagnosed”. (citation Reference No 2).*

**Comment:** P6 Line 9: “However, only six patients underwent pre-treatment Fluoroscopy study”. Was hemodynamic instability the reason for fluoroscopy not done in other patient- reasons can be mentioned.
**Response:** We accept the comment and included the following explanation: *“The remaining patients didn’t undergo fluoroscopy study due to their hemodynamic instability and occasionally due to malfunction of the fluoroscopy machine”* (page-8, lines 154-156).

**Comment:** P6 Line 17-19
“Of the ten patients who received streptokinase, two of them developed fever and one patient developed bleeding as a complication of the treatment”
What type of bleeding complication was encountered? Was it a major or a minor bleed and how was it managed

**Response:** Thank you. One patient developed excessive epistaxis and gum bleeding. Now, we mentioned that in the revised manuscript (page-8, line- 164).

**Comment:** P6 Line 21-23: “The mean value of the mean trans-mitral valve gradient of the patients who had isolated mitral prosthetic stuck valve (12 patients) before therapy was initiated was 18.3mmHG and the post- treatment mean gradient for the group was 7.2mmHG”.
Was the post operative gradients available for these patients. For patients for whom baseline value was not available, what was the criteria used to confirm prosthetic valve obstruction?

**Response:** Thank you for asking. When we say ***“The post treatment gradient”*** we mean the gradient that was measured after receiving the treatment for the stuck valve (it is not the post-operative gradient). Also, when we say ***“The pre-treatment gradient”*** we mean the gradient that was measured before receiving therapy for the stuck valve (at the time of presentation after having the stuck valve). Therefore, we compared the mean gradients of **“before”** and **“after”** receiving treatment for stuck valve. We have revised the mentioned statement to make it understandable (Page-8, line 168).
**Comment:**P6 Line 24-26:
“Two of the patients (patient 4 & 5 and patient 7 & 8) had recurrence of the prosthetic stuck valve. One of them developed a recurrence of the stuck valve three months after the initial episode and the other patient had it three years after the initial episode”
Does this mean 11 patients and 13 episodes- kindly clarify

**Response:** Thank you for pointing out this. We accept the comment and as the reviewer pointed out, there were 11 patients with 13 episodes of stuck valve. We have made the necessary correction in the revised manuscript (page-8, line-170-172 and page9- table-1, page-7, line 133, page-2, line-31).

**Thank you**
